# Antifungal Efficacy of Carbon Quantum Dots Combined with Photodynamic Therapy Against *Alternaria alternata*

**DOI:** 10.3390/foods15183227

**Published:** 2026-09-12

**Authors:** Zhi-Jing Ni, Ruo-Tong Yang, Run-Hui Ma, Wei Wang, Kiran Thakur, Zhao-Jun Wei

**Affiliations:** 1 School of Biological Science and Engineering, Specialty Food Nutrition and Health Innovation Team of Ningxia Hui Autonomous Region, North Minzu University, Yinchuan 750021, China; 2 Ningxia Key Laboratory for the Development and Application of Microbial Resources in Extreme Environments, Yinchuan 750021, China

**Keywords:** carbon quantum dots, *Alternaria alternata*, photodynamic inactivation, ROS, energy metabolism, postharvest disease, antifungal mechanism

## Abstract

*Alternaria alternata*, a major postharvest pathogen of grapes and goji berries, causes black spot disease and produces *Alternaria* toxins that threaten food safety and human health. Carbon quantum dots (CQDs) are emerging promising nanophotosensitizers for photodynamic antimicrobial therapy (PDT), yet their efficacy against *A. alternata* remains unclear. This study systematically investigated the antifungal effect of CDs-PDT against *A. alternata* and revealed the possible mechanisms at cellular and physiological-biochemical levels. The inhibitory effect of CDs-PDT against *A. alternata* exhibited a clear concentration-dependent characteristic, achieving a 100% inhibition rate at 48 h; meanwhile, mycelial dry and fresh weights were reduced by 98.4% and 94.7%, respectively. The possible inhibitory mechanism of CDs-PDT against *A. alternata* was revealed that it damages cell wall and membrane integrity, evidenced by scanning electron microscope (SEM) observation, propidium iodide (PI) staining, increased malondialdehyde (MDA) content, and altered lactate dehydrogenase (LDH) and alkaline phosphatase (AKP) activities. Intracellular reactive oxygen species (ROS) accumulation was confirmed via 2′,7′-dichlorodihydrofluorescein diacetate (DCFH-DA), 3,3′-diaminobenzidine (DAB), and nitroblue tetrazolium (NBT) staining, accompanied by initial upregulation followed by collapse of antioxidant defenses, such as superoxide dismutase (SOD), glutathione peroxidase (GPX), catalase (CAT), glutathione (GSH). CDs-PDT further impaired energy metabolism, reflected by decreased adenosine triphosphate (ATP) and acetyl-CoA content. Additionally, CDs-PDT can also increase *A. alternata*’s sensitivity to exogenous stress. These results indicate that CDs-PDT is an effective strategy for controlling *A. alternata* diseases and provide mechanistic insights into future nanophotosensitizer-based antifungal research in postharvest grape and goji berries management.

## 1. Introduction

*Alternaria* genus is among the most agriculturally destructive plant pathogens worldwide. Among the approximately 300 identified species, *Alternaria alternata* is the most widely distributed, infecting nearly 100 plant species, including vegetables, fruits, and cash crops, causing wilting, postharvest diseases, and causing significant economic losses [1,2]. *A. alternata* is the primary pathogen affecting goji berries during the postharvest period; this pathogen has previously been isolated and identified from infected berries [3]. Beyond direct crop damage, *A. alternata* poses serious health risks, triggering upper respiratory infections and asthma as an allergen [2]. More critically, it secretes a series of *Alternaria* toxins (ATs) with potent carcinogenic and mutagenic effects. For instance, Altertoxin II (ATX II) exhibits at least 50 times greater mutagenicity than Alternariol (AOH) and Alternariol monomethyl ether (AME) [4]. Because these toxins remain in the food even after visible spoilage has been controlled, contamination by *Alternaria* poses a dual threat to agricultural productivity and food safety [5,6].

So far, conventional management of *A. alternata* relies heavily on synthetic fungicides such as pyraclostrobin and azoxystrobin as the primary control measure [7]. However, their continued use raises concerns regarding environmental hazards, fungicide residues, and the accelerating emergence of resistant fungal strains [8]. These limitations have intensified interest in eco-friendly, multi-target antifungal alternatives that are less susceptible to resistance development.

Carbon dots (CDs) have emerged as a promising class of zero-dimensional carbon-based nanomaterials (<10 nm). Given their excellent optical properties and low cytotoxicity, they are well-suited for applications in the biomedical and antimicrobial fields [9]. According to their structural and compositional characteristics, CDs are classified into carbon quantum dots (CQDs), graphene quantum dots (GQDs), carbon nanodots (CNDs), and polymer dots (PDs) [10,11,12].

CDs have demonstrated broad-spectrum antimicrobial activity against various bacterial and fungal pathogens, including *Staphylococcus aureus*, *Escherichia coli*, *Aspergillus* spp., and *Candida albicans* [13,14,15,16,17]. Their antimicrobial mechanisms are multifaceted, including electrostatic adsorption onto negatively charged microbial surfaces, hydrophobic interaction-mediated membrane disruption, DNA fragmentation, and reactive oxygen species (ROS) generation [18,19,20,21,22]. It is worth noting that CDs also exhibit concentration-dependent phototoxicity, which occurs through physical interactions with the cell surface and can take place even without light activation [23]. This multi-target mechanism of action distinguishes CDs from traditional antibiotics and significantly reduces the possibility of resistance evolution [24].

A particularly promising application of CDs lies in photodynamic therapy (PDT), wherein a photosensitizer absorbs light energy and generates cytotoxic ROS through Type I (electron/hydrogen transfer, yielding hydroxyl radicals and superoxide anions) or Type II (energy transfer to molecular oxygen, yielding singlet oxygen) photochemical pathways [25].

Blue light-emitting diodes (LEDs) excite endogenous photosensitizers such as porphyrins, generating oxidative damage sufficient to inhibit pathogen viability [26]. Blue LED irradiation alone has been shown to significantly inhibit spore germination and hyphal elongation in postharvest pathogens such as *Colletotrichum acutatum*, *Botrytis cinerea*, and *Penicillium digitatum* [27,28]. However, PDT efficacy using endogenous photosensitizers is highly dependent on fruit surface topography; smooth-surfaced produce (cucumbers, tomatoes) shows a better effect than structurally complex surfaces such as strawberries [29]. This limitation has allowed the incorporation of exogenous photosensitizers (curcumin and riboflavin), which enhance ROS generation effectively without chemical residues [30]. Given their strong light-absorbing properties and biocompatibility, CDs, as effective photosensitizers, show promise as compelling next-generation exogenous photosensitizers for PDT-based postharvest preservation.

Our research group has previously demonstrated CDs-PDT potential in postharvest preservation, including reduced grape senescence via antioxidant regulation [31] and improved goji berry shelf life through metabolic modulation [32]. Nevertheless, these previous studies mainly focused on the macroscopic fruit preservation performance, and the underlying antifungal action against causal pathogens was not further clarified. Therefore, we are committed to exploring the unexplored specific antifungal mechanisms against *A. alternata*, the primary goji berry pathogen. To our knowledge, this is the first-ever study to systematically demonstrate CDs-PDT antifungal efficacy against *A. alternata* while establishing an integrated mechanistic framework spanning cell wall/membrane damage, intracellular ROS dynamics, antioxidant system regulation, and energy metabolism impairment, elucidating its underlying mechanisms at both cellular and physiological-biochemical levels. This work aims to provide direct experimental evidence and theoretical support for the broader application of CDs-PDT as an eco-friendly antifungal strategy in postharvest agricultural preservation.

## 2. Materials and Methods

### 2.1. Strains and Reagents

*A. alternata* strain was previously isolated from infected goji berries (*Lycium barbarum* L.) and identified by morphological and molecular methods [3], and kindly provided by the Laboratory of Specialty Food Development and Utilization at North Minzu University. The strain was preserved at 4 °C on potato dextrose agar (PDA) slants and subcultured monthly. Ascorbic acid-derived carbon dots (CDs) were synthesized and characterized (transmission electron microscopy, Fourier-transform infrared spectroscopy, UV-Vis absorption, and fluorescence spectroscopy) as previously described [33]. All reagents were of analytical grade and purchased from Sigma-Aldrich (St. Louis, MO, USA) unless otherwise specified.

### 2.2. Antifungal Activity of Carbon Dots Against A. alternata

#### 2.2.1. In Vitro Screening of the Antifungal Activity of Carbon Dots Against *A. alternata*

To comprehensively evaluate the in vitro antifungal activity of CDs against *A. alternata* and to distinguish the effects of blue light and the combined treatment of CDs and blue light on the growth of *A. alternata*, this study employed a completely randomized factorial design. CDs solutions with concentrations of 5, 10, 15, 20, 25, and 30 mg/mL were prepared using sterile water. *A. alternata* cakes with a diameter of 5 mm were thoroughly immersed in the CDs solutions. A blue LED light source with a wavelength of 465 nm was used, with an irradiance of 145 mW/cm^2^ for 30 min to form the CDs in the synergistic phototreatment group. The group without light exposure served as the CDs group. Sterile water without CDs was subjected to the same treatment protocols with and without light exposure, serving as the phototreatment group and the blank control group, respectively. The treated *A. alternata* cakes were inoculated onto PDA medium and incubated in the dark at 28 °C for 48 h; colony diameters were then measured using the cross-counting method. The experiment was conducted in triplicate. The mycelial growth inhibition rate was calculated using Equation 1. A growth inhibition curve was plotted with CDs concentration on the *x*-axis and growth inhibition rate on the *y*-axis, and the CDs concentration at which 50% inhibition was observed (IC_50_) was calculated.

(1) $$\mathrm{Inhibition}\;\mathrm{rate}(\%)=\frac{C-T}{C-5}\;\times \;100\%$$

Here, C represents the colony growth diameter in the blank control group, and T represents the colony growth diameter in the CDs synergistic light treatment group and the CDs group.

#### 2.2.2. Effect of Carbon Dots on Biomass Accumulation in *A. alternata*

The effects of CDs on biomass accumulation in *A. alternata* were evaluated using fresh weight and dry weight of mycelium as indicators. Following the method described in Section 2.2.1, CD solutions at concentrations of 0 and 18 mg/mL were prepared for dark and light treatments of *A. alternata*. The fungus was inoculated onto Potato Dextrose Broth (PDB) medium and cultured in the dark at 28 °C and 160 r/min for 24 h and 48 h (the group with no CDs and no light exposure was designated as CK−, the group with CDs and no light exposure was designated as I−, the group without CDs under light was designated as CK+, and the group with CDs under light was designated as I+, subsequent experimental groups were labeled in the same manner). Mycelia were collected by filtering through four layers of gauze, moisture was blotted dry with absorbent paper, and the weight was recorded as the fresh weight of the mycelia; the mycelia were then freeze-dried to constant weight to obtain the dry weight of the mycelia. The experiment was conducted with three biological replicates.

### 2.3. Effects of Carbon Dots on the Cell Wall of A. alternata

#### 2.3.1. Scanning Electron Microscopy Observation of the Effects of CDs on *A. alternata* Hyphae

The effects of CDs on the physical structure of the fungal hyphae were observed using a scanning electron microscope (SEM). Following the method described in Section 2.2.1, 18, 25, and 30 mg/mL CD solutions were prepared for light treatment of *A. alternata*, with sterile water in the absence of light serving as the control. The cultures were inoculated onto PDA and incubated for 48 h. The mycelia were collected, and samples were prepared according to SEM sample preparation procedures, then observed and photographed under the scanning electron microscope (JEOL, Peabody, MA, USA). The experiment was conducted with three biological replicates.

#### 2.3.2. Effects of Carbon Dots on Cell Wall Components of *A. alternata*

Calcofluor White (CFW) is a white fluorescent dye that specifically binds to chitin and β-1,3 and β-1,4 polysaccharides in the cell wall, emitting a bluish-white fluorescence under a fluorescence microscope; its fluorescence intensity directly reflects cell wall integrity and chitin content [34]. Mycelia were treated and collected as described in Section 2.3.1. Samples were stained with 10 µg/mL CFW (Shanghai Mcline Biochemical Technology Co., Ltd., Shanghai, China) in 10 mM phosphate-buffered saline (PBS, pH 7.4) for 30 min at room temperature in the dark. After washing three times with phosphate buffer, hyphae were observed under a fluorescence microscope (Olympus CKX53, Tokyo, Japan) with excitation at 365 nm and emission at 435 nm. Three biological replicates were performed.

#### 2.3.3. Effect of Carbon Dots on Alkaline Phosphatase Activity in *A. alternata*

Alkaline phosphatase (AKP) is primarily found between the fungal cell membrane and the cell wall; measuring intracellular AKP activity can therefore indirectly reflect cell wall integrity [35]. The fungal cake was processed as described in Section 2.2.2, inoculated onto PDB medium, and the mycelium was harvested after 24 and 48 h of incubation. Enzyme activity was determined in accordance with the instructions for the AKP Activity Assay Kit (Suzhou Keming Biotechnology Co., Ltd., Suzhou, China). The experiment was conducted with three biological replicates.

### 2.4. Effects of Carbon Dots on the Cell Membrane of A. alternata

#### 2.4.1. Analysis of the Effects of CDs on the Cell Membrane of *A. alternata* Using the Propidium Iodide Staining Method

Propidium iodide (PI) is a red, fluorescent dye that stains dead cells. It cannot penetrate the cell membranes of living cells, but it can pass through damaged cell membranes, where it binds to DNA and emits red fluorescence under a fluorescence microscope, thereby indicating damage to the cell membrane [36,37]. Mycelia were treated and collected as described in Section 2.3.1. Samples were washed three times with 10 mM PBS and stained with 5 µg/mL PI for 15 min at room temperature in the dark. After three additional washes with 10 mM PBS to remove excess dye, hyphae were observed under a fluorescence microscope with excitation at 535 nm and emission at 617 nm. Three biological replicates were performed.

#### 2.4.2. The Effect of Carbon Dots on Malondialdehyde Levels and Lactate Dehydrogenase Activity in *A. alternata*

In order to evaluate the effect of CDs in combination with blue light treatment on the level of lipid peroxidation in *A. alternata* cell membranes, the thiobarbituric acid (TBA) method was used to determine malondialdehyde (MDA) levels. As the end product of membrane lipid peroxidation, the concentration of MDA can indirectly reflect the extent of oxidative damage to cell membranes caused by ROS [38,39]. Lactate dehydrogenase (LDH) is a key enzyme in the glycolytic pathway. It is normally present within cells and cannot cross the cell membrane; damage to the cell membrane leads to the leakage of intracellular LDH and a reduction in its intracellular activity, thereby indirectly reflecting the integrity of the cell membrane [40]. The samples were processed as described in Section 2.2.2 and mycelia were collected at 24 h and 48 h. Measurements were carried out in accordance with the instructions for the MDA content assay kit and the LDH activity assay kit (Suzhou Keming Biotechnology Co., Ltd., Suzhou, China). The experiment was conducted with three biological replicates.

### 2.5. Effect of Carbon Dots on the Steady State of Reactive Oxygen Species in A. alternata

#### 2.5.1. Effect of Carbon Dots on ROS Production in *A. alternata*

2′,7′-dichlorodihydrofluorescein diacetate (DCFH-DA) is not fluorescent in itself, it freely crosses the cell membrane and, once inside the cell, is hydrolysed by intracellular esterases to form 2′,7′-dichlorodihydrofluorescein (DCFH), whereas DCFH cannot cross the cell membrane, it remains within the cell and is oxidised by ROS to form 2′,7′-dichlorofluorescein (DCF), which emits green fluorescence, thereby indirectly reflecting the intracellular ROS levels [41]. 3,3′-diaminobenzidine tetrahydrochloride (DAB) and nitroblue tetrazolium (NBT) are oxidised by hydrogen peroxide (H_2_O_2_) and the superoxide anion (O_2_^−^) within fungal cells, respectively, to form reddish-brown and blue/purple formazan precipitates. Following staining, changes in colour are observed under a biological microscope (E5, Ningbo Yongxin Optics Co., Ltd., Ningbo, China) to reflect the oxidative stress experienced by the cells [42,43]. The hyphae were processed and treated as described in Section 2.3.1. They were collected and washed after 24 h, stained in a 10 μM DCFH-DA solution at room temperature in the dark for 30 min, washed again, and then observed and photographed under a fluorescence microscope. For DAB staining (detecting H_2_O_2_) and NBT staining (detecting O_2_^−^), the Plant Tissue Reactive Oxygen Species Detection Kit (Beijing Suolaibao Technology Co., Ltd., Beijing, China) was used according to the manufacturer’s instructions, with samples observed under a light microscope (Olympus CKX53, Tokyo, Japan). H_2_O_2_ content was determined in accordance with the instructions for the H_2_O_2_ Content Assay Kit (Suzhou Keming Biotechnology Co., Ltd., Suzhou, China). The experiment was conducted with three biological replicates.

#### 2.5.2. Effects of Carbon Dots on the Antioxidant System of *A. alternata*

Superoxide Dismutase (SOD) is the first line of defense in the cell’s antioxidant defense system, catalyzing the conversion of O_2_^−^ into H_2_O_2_ and molecular oxygen (O_2_). Catalase (CAT) breaks down the H_2_O_2_ produced by SOD into H_2_O and O_2_, while glutathione peroxidase (GPX) and reduced glutathione (GSH) also exert synergistic antioxidant effects. The activity of these enzymes, the levels of these compounds, and the total antioxidant capacity can reflect the status of the cellular antioxidant system [44]. Sample preparation was performed as described in Section 2.2.2. Mycelia were collected at 24 h and 48 h, and enzyme activity was determined according to the instructions for the SOD, CAT, and GPX enzyme activity assay kits (Suzhou Keming Biotechnology Co., Ltd., Suzhou, China). GSH content and total antioxidant capacity were also determined according to the instructions for the respective kits (Suzhou Keming Biotechnology Co., Ltd., Suzhou, China). The experiment was conducted with three biological replicates.

### 2.6. Effects of Carbon Dots on the Energy Metabolism of A. alternata

Adenosine triphosphate (ATP) is the cell’s “powerhouse,” and acetyl-CoA is the starting substance of the tricarboxylic acid cycle; changes in the levels of these two substances directly reflect the cell’s energy metabolism status [45,46]. Samples were prepared as described in Section 2.2.2. Mycelia were collected at 24 h and 48 h and analyzed according to the instructions for the ATP Assay Kit (Suzhou Keming Biotechnology Co., Ltd., Suzhou, China) and the Acetyl-CoA Assay Kit (Beijing Solabio Technology Co., Ltd., Beijing, China). The experiment was conducted with three biological replicates.

### 2.7. Effects of Carbon Dots on the Stress Sensitivity of A. alternata

The mycelia were processed according to the method described in Section 2.3.1, then inoculated separately onto PDA medium and into the center of PDA medium containing 1 M NaCl, 1 M sorbitol, 0.01% sodium dodecyl sulfate (SDS), 3 mM H_2_O_2_, and 100 μM Congo red (CR). The plates were incubated at a constant temperature of 28 °C in the dark for 3, 5, and 7 d. Colony diameters were recorded using a cross-counting method, and photographs were taken for documentation. The experiment was conducted with three biological replicates.

### 2.8. Data and Analysis

All data are presented as mean ± standard deviation (SD) from three independent biological replicates (n = 3). Statistical significance was determined by one-way analysis of variance (ANOVA) followed by Tukey’s honestly significant difference (HSD) post-hoc test for multiple comparisons, using SPSS software (version 26.0, IBM, Armonk, NY, USA). *p* < 0.05 was considered statistically significant. Figures were generated using GraphPad Prism (version 10.0, GraphPad Software, San Diego, CA, USA).

## 3. Results

### 3.1. The Combination of CDs and PDT Treatment Significantly Inhibits the Growth of A. alternata

**Figure 1:**
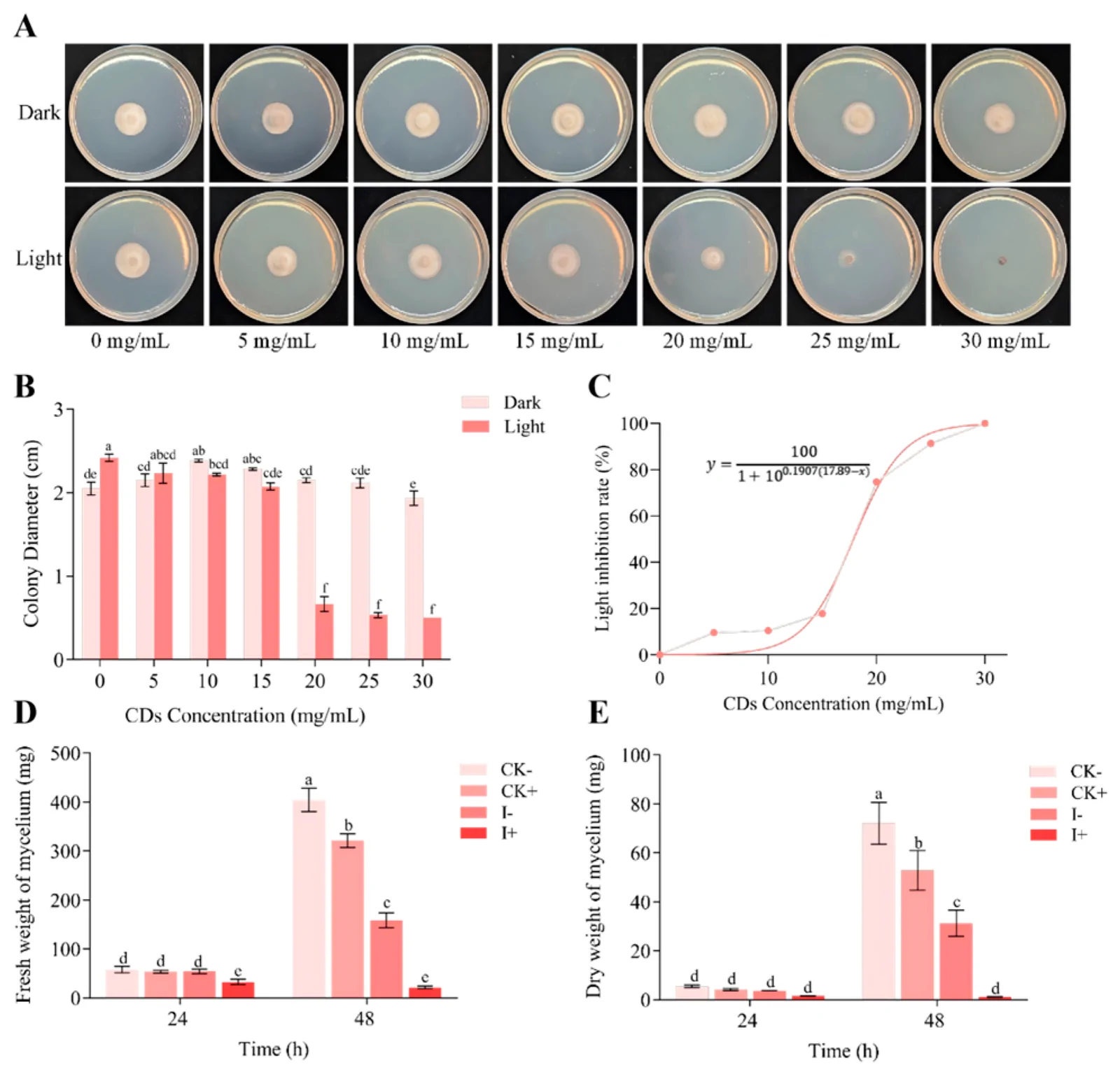
Antifungal activity of CDs against *A. alternata*. Effects of CD solutions at different concentrations on *A. alternata* (**A**); diameters of *A. alternata* colonies after treatment (**B**); hyphal growth inhibition rates (**C**); dry weight (**D**) and fresh weight (**E**) of hyphae. Data are presented as mean ± SD (n = 3). Different letters indicate significant differences (*p* < 0.05, one-way ANOVA followed by Tukey’s HSD test).

Inhibiting the growth of plant pathogenic fungi is fundamental to controlling postharvest diseases [47]. Under dark conditions, compared with the 0 mg/mL CDs group, there were no significant changes in the colony diameters of *A. alternata* on PDA medium after treatment with 5, 10, 15, 20, 25, and 30 mg/mL CDs (Figure 1A,B). After combined treatment with CDs-PDT, compared with the 0 mg/mL CDs group, the colony diameters of *A. alternata* on PDA medium treated with 5, 10, and 15 mg/mL CDs were slightly reduced. But the colony diameters of *A. alternata* on PDA medium treated with 20, 25 and 30 mg/mL CDs decreased significantly by 72%, 76%, and 80%, respectively, with the reduction becoming more pronounced as the CDs concentration increased (Figure 1A,B). The inhibition rate under the combined treatment of CDs-PDT was calculated using a formula. A growth inhibition curve was plotted with CDs concentration on the *x*-axis and inhibition rate on the *y*-axis, and the IC_50_ was calculated to be 17.89 mg/mL (Figure 1C) using non-linear regression analysis. Based on this, 18 mg/mL was selected as the working concentration for all subsequent mechanistic experiments.

Regarding biomass accumulation, after 24 h of cultivation, there were no significant changes in fresh weight in the CK−, CK+, and I− groups compared with the CK− group, but fresh weight in the I+ group decreased by 43.8%. However, there were no significant changes in dry weight among the CK−, CK+, I−, and I+ groups. After 48 h of cultivation, compared with the CK− group, fresh weights in the CK+, I−, and I+ groups decreased by 20.5%, 60.8%, and 94.7%, respectively, and dry weights decreased by 26.6%, 40.8%, and 98.4%, respectively (Figure 1D,E). In summary, both blue-light LEDs and CDs exhibit some inhibitory effects on *A. alternata*. However, the combination of CDs and PDT treatment demonstrated the most effective inhibition of *A. alternata*. Notably, the significant inhibition (40.8% dry weight reduction) observed with CDs alone (I− group) at 48 h indicates intrinsic dark toxicity of CDs, likely due to physical membrane disruption and surface interactions, as previously reported for other carbon nanomaterials, where surface charge and functional groups can directly induce oxidative stress and membrane damage even without photoactivation [23]. This suggests that neither the photosensitizer alone nor light exposure is the primary factor in antifungal activity. Rather, the observed and measured antifungal effects primarily result from the synergistic effect of CDs and PDT, which is consistent with the previous studies [48,49]. Specifically, the initial physical interaction of CDs with the fungal cell wall may compromise membrane integrity, thereby enhancing photosensitizer penetration and potentiating the photodynamic reaction—a cooperative mechanism analogous to strategies that overcome biological barriers to improve photodynamic efficacy [49].

### 3.2. Effects of PDT Combined with CDs on the Cell Wall of A. alternata

**Figure 2:**
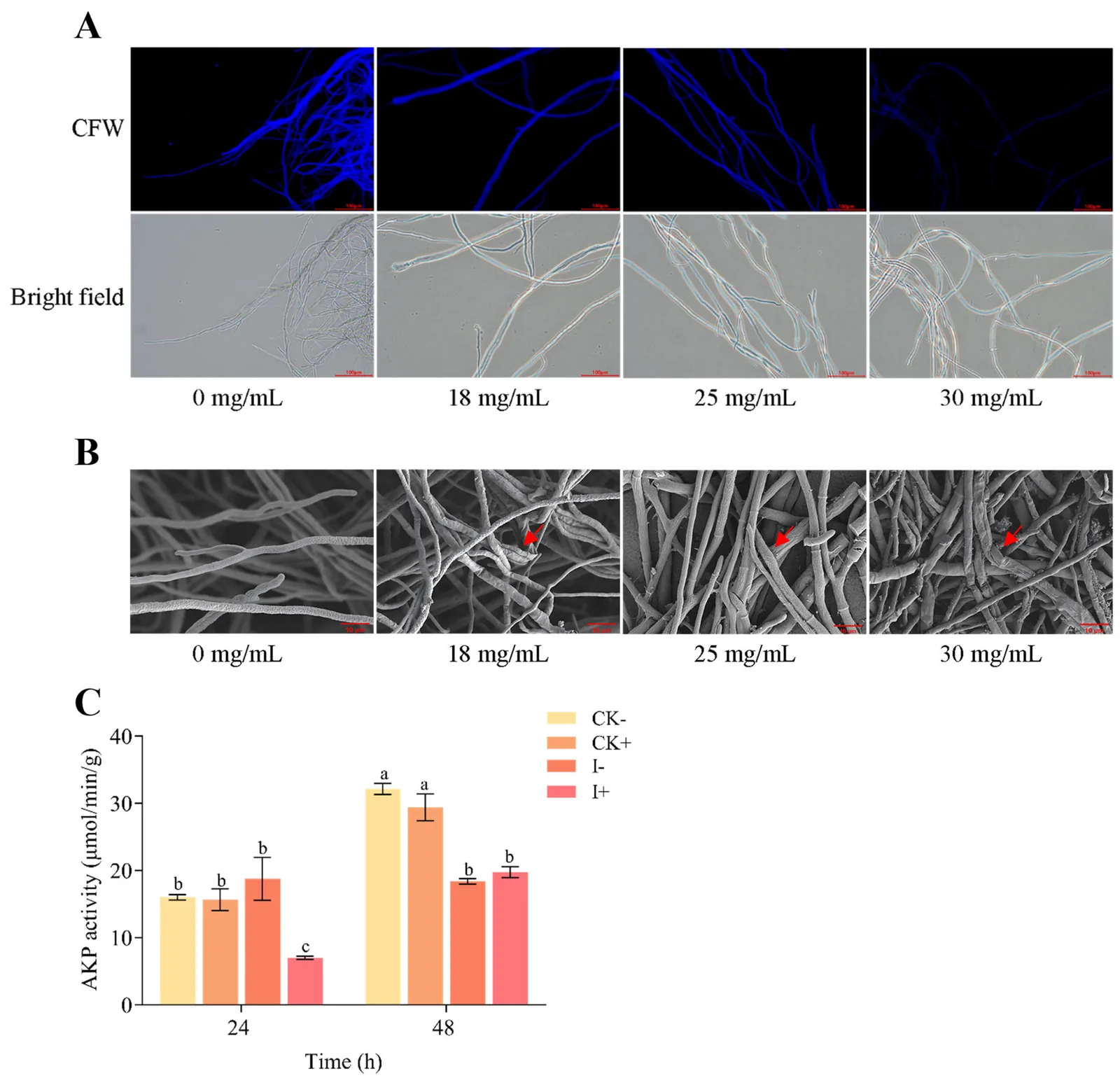
Effects of CDs on the cell wall of *A. alternata*. CFW staining (**A**); ultrastructural observation (**B**); intracellular AKP enzyme activity (**C**). Data are presented as mean ± SD (n = 3). Different letters indicate significant differences (*p* < 0.05, one-way ANOVA followed by Tukey’s HSD test).

The fungal cell wall consists of a chitin skeleton, which is essential for cell integrity and environmental adaptation [50]. After CFW staining, observation under a fluorescence microscope revealed that, compared with the 0 mg/mL CDs group, the blue fluorescence of *A. alternata* hyphae became weaker as the CDs concentration increased (18, 25, 30 mg/mL) (Figure 2A), indicating that the chitin or glucan components of the cell wall decreased with increasing CDs concentration. At the same time, SEM revealed that the hyphae in the 0 mg/mL CDs group had smooth, intact surfaces; as the CDs concentration increased, the surfaces of the *A. alternata* hyphae exhibited increasingly severe wrinkling and twisting (Figure 2B). Assessment of intracellular AKP enzyme activity in *A. alternata* cells showed that, after 24 h of culture, there were no significant changes in AKP enzyme activity compared with CK−, CK+, and I−. However, I+ enzyme activity decreased by 56.3%. After 48 h of culture, compared with CK−, there was no significant change compared with CK+, but I− and I+ enzyme activities decreased by 42.8% and 38.5%, respectively (Figure 2C). Combined with CFW staining and SEM results, this indicates that the leakage of intracellular AKP leads to a decrease in enzyme activity. The results indicate that the combined use of PDT technology and CDs led to the disruption of the cell wall structure of *A. alternata*, resulting in the disruption of the cell wall structure and the leakage of intracellular AKP. This is likely due to the fact that, under the photodynamic effect, CDs generate ROS that oxidize the chitin and glucan polysaccharide frameworks in the cell wall, disrupting their cross-linked structure [51], thereby causing the cell wall to lose its physical barrier function, as evidenced by weakened CFW fluorescence, wrinkling of the hyphal surface, and AKP leakage. Jan [52] et al. found that PDT mediated by hypocrellin B caused significant damage to the cell wall of C. albicans and suggested that ROS may be responsible for the damage to cytoplasmic and cell wall components, which is consistent with our conclusions.

### 3.3. Effects of PDT Combined with CDs on the Cell Membrane of A. alternata

**Figure 3:**
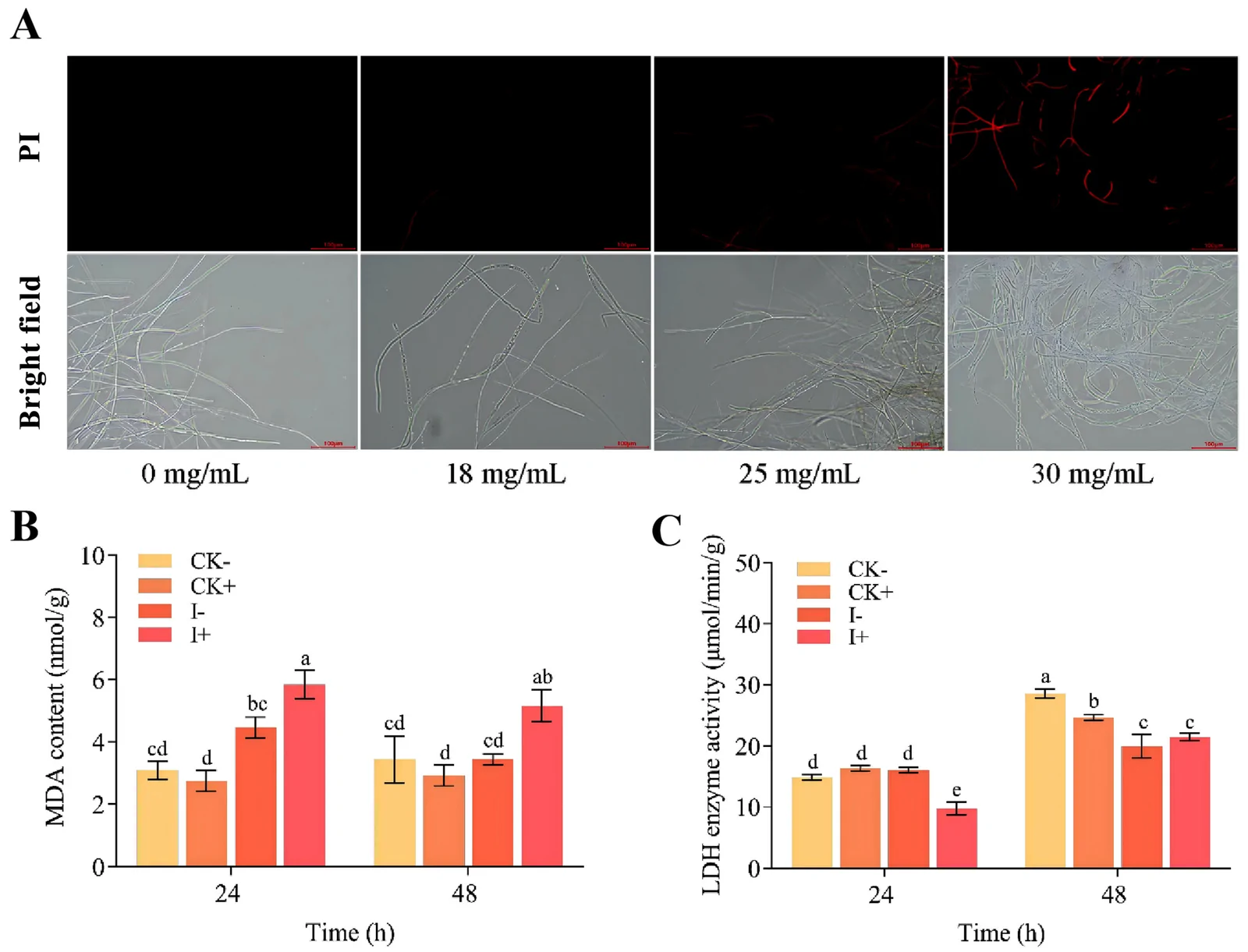
Effects of CDs on the cell membranes of *A. alternata*. PI staining (**A**); MDA content (**B**); LDH activity (**C**). Different letters indicate significant differences (*p* < 0.05, one-way ANOVA followed by Tukey’s HSD test).

PI is a nucleic acid fluorescent dye that cannot penetrate intact cell membranes. When the cell membrane is damaged, it enters the cell, binds to DNA, and emits red fluorescence. Therefore, the stronger the red fluorescence from PI staining, the more severe the membrane damage [36,37]. Following PI staining, compared with the 0 mg/mL CDs group, the red fluorescence of *A. alternata* hyphae became brighter as the CDs concentration increased (18, 25, 30 mg/mL) (Figure 3A), indicating impaired cell membrane integrity. MDA is the end product of membrane lipid oxidation and its concentration reflects oxidative damage to cell membrane lipids [38]. The accumulation of ROS leads to the buildup of lipid peroxides, which in turn generate MDA, causing cytotoxicity, cell lysis, and even cell death [39]. In this study, after 24 h of culture, there was no significant difference in MDA levels between CK− and CK+; MDA levels in I− and I+ increased by 44.4% and 88.9%, respectively. After 48 h of culture, there were no significant changes in MDA levels in CK− and CK+, nor in I−, but MDA levels in I+ increased by 50% (Figure 3B). LDH is a cytoplasmic metabolic enzyme; a decrease in its intracellular activity or an increase in its extracellular activity can indirectly reflect the integrity of the cell membrane [40]. When LDH enzyme activity in *A. alternata* cells was measured, after 24 h of incubation, there were no significant changes in enzyme activity compared with CK−, CK+, and I−. However, LDH enzyme activity in I+ decreased by 34.4%. After 48 h of incubation, compared with CK−, enzyme activity in the CK+, I−, and I+ groups decreased by 13.7%, 29.9%, and 24.8%, respectively. In summary, the enhanced red fluorescence of PI, increased MDA levels, and decreased intracellular LDH activity indicate that the combination of CDs-PDT may cause oxidative damage to the membrane structure through ROS-mediated lipid peroxidation, leading to the leakage of intracellular contents and resulting in irreversible damage to the cell membranes of *A. alternata*.

### 3.4. Effects of PDT Combined with CDs on the ROS Steady State of A. alternata

**Figure 4:**
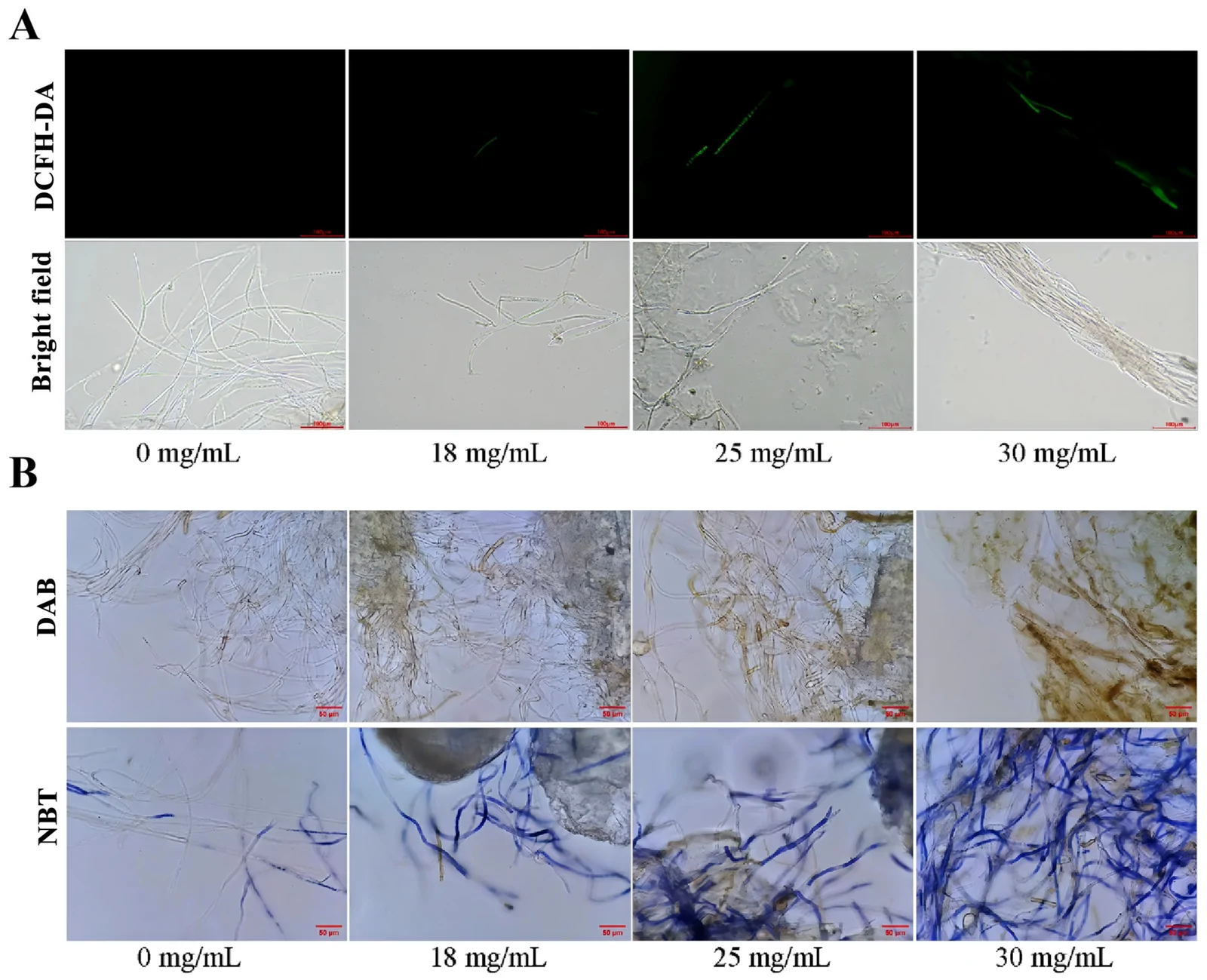
Effect of CDs on ROS production by *A. alternata*. DCFH-DA staining results (**A**); DAB and NBT staining results (**B**).

**Figure 5:**
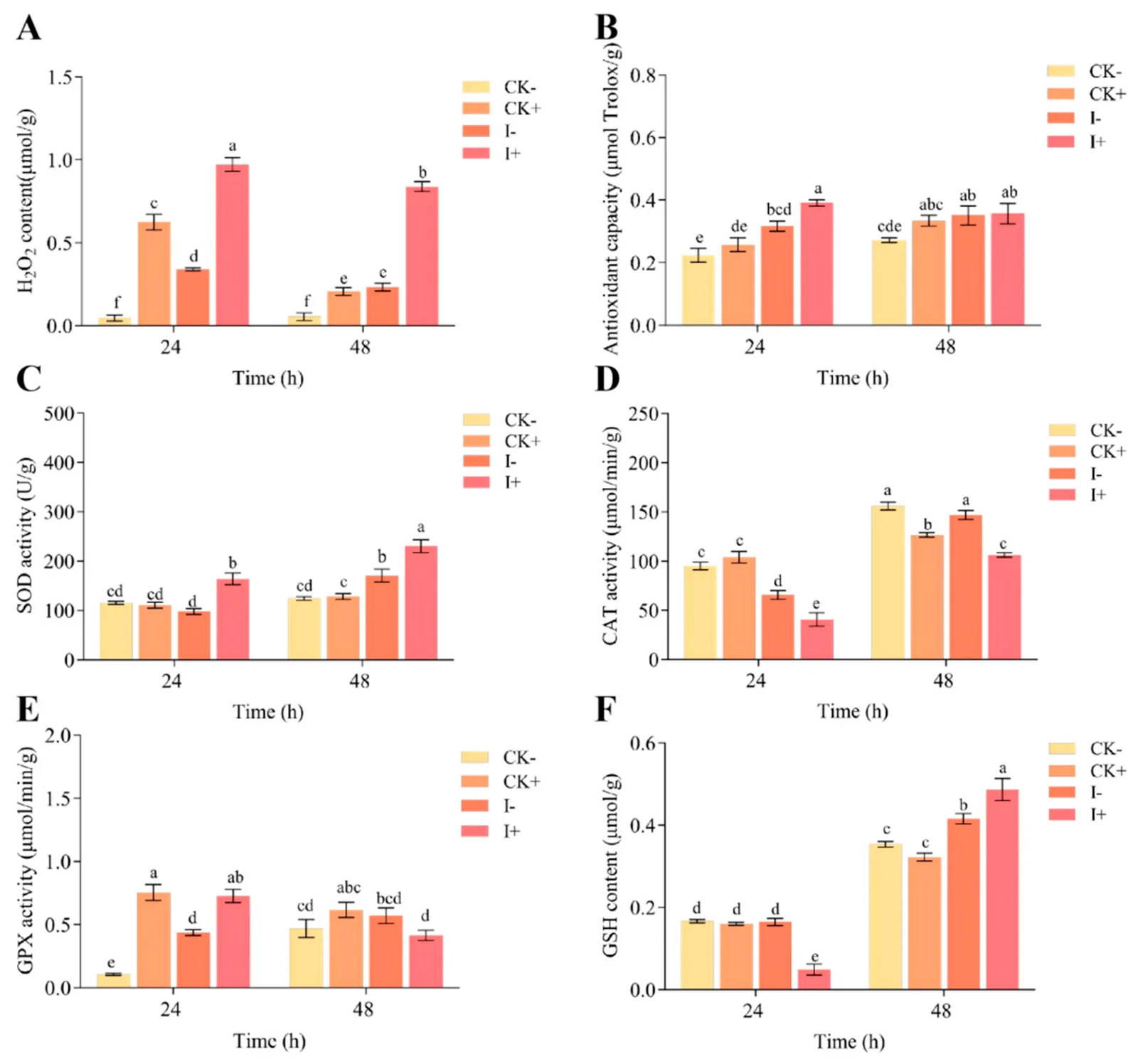
Effects of CDs on ROS production and the antioxidant system in *A. alternata*. H_2_O_2_ content (**A**); total antioxidant capacity (**B**); SOD activity (**C**); CAT activity (**D**); GPX activity (**E**); GSH activity (**F**). Data are presented as mean ± SD (n = 3). Different letters indicate significant differences (*p* < 0.05, one-way ANOVA followed by Tukey’s HSD test).

The homeostasis of intracellular ROS is crucial for normal cellular metabolism. Excessive levels of ROS can attack biomacromolecules such as proteins, lipids, and DNA, leading to oxidative damage and even cell death [53]. After staining with the DCFH-DA probe, observation under a fluorescence microscope revealed that, compared with the 0 mg/mL CDs group, the green fluorescence of *A. alternata* hyphae became brighter as the CDs concentration increased (18, 25, 30 mg/mL) (Figure 4A), indicating that ROS also accumulated continuously as the concentration increased. After DAB and NBT staining, compared with the 0 mg/mL CDs group, the 18, 25, and 30 mg/mL CDs groups showed a greater number of brown and blue hyphae under a light microscope, respectively. Furthermore, as the treatment concentration increased, the proportion of brown and blue hyphae also increased (Figure 4B), indicating that the accumulation of H_2_O_2_ and O_2_^−^, combined with the CDs-PDT treatment, led to oxidative stress in *A. alternata*. H_2_O_2_ is the primary ROS within cells; it is not only a byproduct of oxidative metabolism but also an important signaling molecule. Excessive H_2_O_2_ can disrupt normal cellular metabolism [53]. Analysis of intracellular H_2_O_2_ levels revealed that, compared with CK−, the H_2_O_2_ levels in CK+, I−, and I+ increased by 12.5 times, 6.3 times, and 20 times, respectively, after 24 h of culture, and by 2.7 times, 3.2 times, and 14.2 times, respectively, after 48 h of culture (Figure 5A). Correspondingly, when total antioxidant capacity was measured, it was found that, compared with CK−, there were no significant changes in the CK+ group after 24 h and 48 h of culture. However, the antioxidant capacities of the I− and I+ groups increased by 41.9%, 74.9%, 29.2%, and 31.3%, respectively (Figure 5B). Compared with CK−, after 24 h of culture, there were no significant changes in SOD activity in the CK+ and I− groups, but the enzyme activity in the I+ group increased by 42.4%. After 48 h of culture, compared with CK−, there was no significant change in SOD activity in the CK+ group, but the enzyme activities of I− and I+ increased by 36.9% and 84.8%, respectively (Figure 5C). These results indicate that cells activated the response mechanism of the antioxidant defense system under ROS stress. Compared with CK−, there was no significant change in CAT enzyme activity in CK+ after 24 h of culture, while I− and I+ decreased by 30.9% and 57.1%, respectively. After 48 h of culture, compared with CK−, there was no significant change in I−, while CK+ and I+ decreased by 18.8% and 31.9%, respectively (Figure 5D). This suggests that CAT is more susceptible to ROS-mediated inactivation than SOD or GPX, likely due to the presence of a heme group at its active site, which is vulnerable to oxidative modification. The 20-fold increase in H_2_O_2_ at 24 h (Figure 5A), combined with 57.1% CAT inhibition, creates a positive feedback loop, accelerating oxidative damage.

GPX enzyme activity and GSH levels serve as key enzymes and compounds for the synergistic scavenging of ROS. GPX uses GSH as an electron donor to catalyze the reduction of H_2_O_2_ and organic peroxides, while GSH, as the most abundant non-enzymatic antioxidant in cells, plays a critical role in maintaining cellular redox balance [44]. After 24 h of incubation, GPX activity in the CK+, I−, and I+ groups increased by 6.1 times, 3.1 times, and 5.8 times, respectively, compared with the CK− group. After 48 h of incubation, there were no significant changes in GPX activity in the CK+, I−, and I+ groups compared with the CK− group (Figure 5E). The return of GPX activity to baseline at 48 h suggests enzymatic exhaustion or protein oxidation rather than transcriptional downregulation.

In terms of GSH content, there were no significant changes in CK+ and I− compared with CK−, but I+ decreased by 70.7%. After 48 h of culture, there were no significant changes in CK+ compared with CK−, while I− and I+ increased by 17.6% and 37.7%, respectively. The extensive GSH depletion (70.7%) at 24 h in the I+ group, followed by recovery at 48 h, suggests that GSH was consumed in the early response to the ROS burst, followed by compensatory synthesis.

In summary, following treatment with CDs combined with PDT, the green fluorescence of DCFH-DA was significantly enhanced, and the ratio of brown (DAB) to blue (NBT) hyphae increased, directly indicating a burst of total intracellular ROS. The H_2_O_2_ content was 20 times higher than that in the control (CK); this data further confirms the rapid accumulation of ROS. The activities of the antioxidant systems SOD and GPX increased, but a significant decrease in CAT activity led to impaired H_2_O_2_ clearance. GSH was extensively depleted in the early stages, ultimately causing the collapse of the cellular antioxidant defense system and the irreversible disruption of ROS homeostasis. This is generally consistent with the findings of Wang [54] et al., who reported that exogenous L-arginine activates ROS production in *A. alternata* and induces an upregulation of SOD, CAT, and GPX activity and gene expression.

### 3.5. Effects of PDT Combined with CDs on the Energy Metabolism of A. alternata

**Figure 6:**
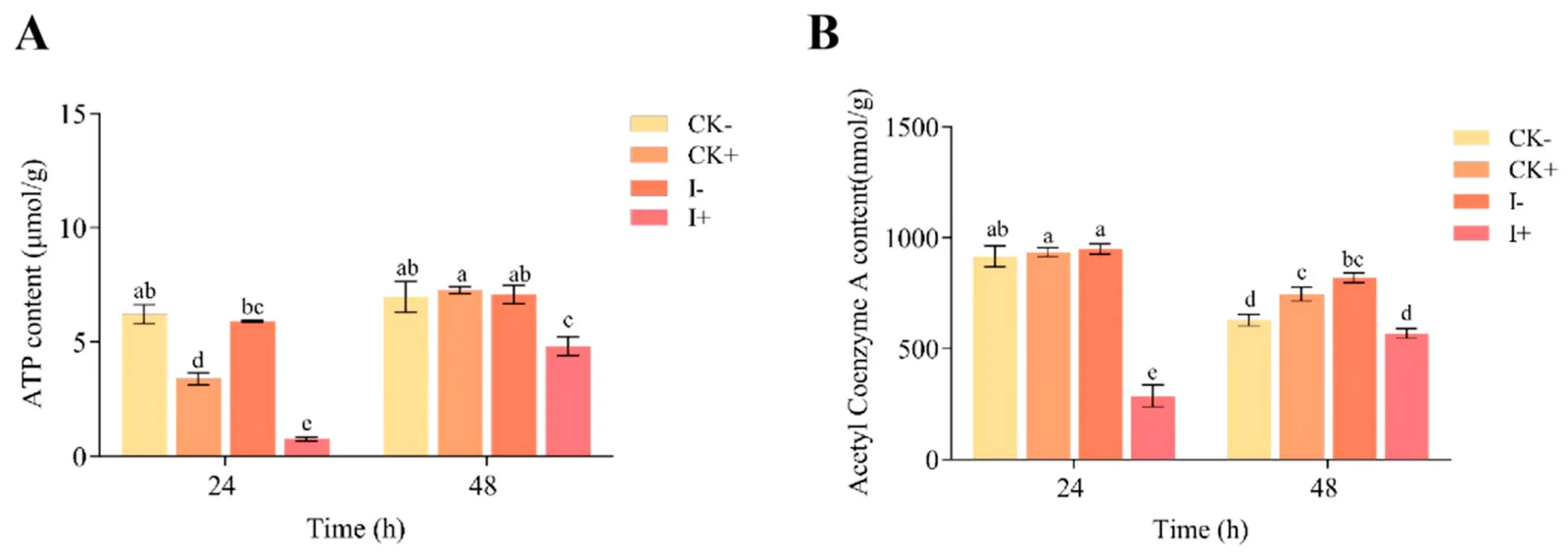
Effects of CDs on the energy metabolism of *A. alternata*. ATP content (**A**); Acetyl Coenzyme A content (**B**). Different letters indicate significant differences (*p* < 0.05, one-way ANOVA followed by Tukey’s HSD test).

ATP is the energy currency of cells and ATP levels directly reflect a cell’s energy metabolism [45]. In this study, after 24 h of culture, ATP levels in the I− group showed no significant change compared with the CK− group, while those in the CK+ and I+ groups decreased by 45.5% and 87.8%, respectively. After 48 h of culture, ATP levels in the CK+ and I− groups showed no significant change compared with the CK− group, but those in the I+ group decreased by 30.6% (Figure 6A). Acetyl-CoA is the starting substrate of the tricarboxylic acid cycle (TCA cycle). Once acetyl-CoA enters the TCA cycle, it undergoes a series of oxidative reactions to produce nicotinamide adenine dinucleotide (NADH) and flavin adenine dinucleotide (FADH_2_), which provide the reducing power required for oxidative phosphorylation to drive ATP synthesis [46,55]. After 24 h of incubation, there were no significant changes compared with the CK−, CK+, and I− groups. However, the acetyl-CoA content in the I+ group decreased by 68.7%. After 48 h of incubation, the CK−, CK+, and I− groups increased by 18.6% and 30.4%, respectively, compared with the CK− group, while the I+ group showed no significant change (Figure 6B). Reduced acetyl-CoA levels are probably caused by oxidative inactivation of important TCA cycle enzymes, including α-ketoglutarate dehydrogenase and pyruvate dehydrogenase, both of which have redox-sensitive thiol groups. In a study comparing the energy metabolism of PDT photosensitizers and photoactivated chemotherapeutic agents, Mitchell [56] and colleagues concluded that acute treatment of cells with PDT agents immediately inhibits the mitochondrial respiratory chain, which ultimately results in impaired ATP synthesis. According to research, oxidative stress can directly inhibit important TCA cycle enzymes, which lowers ATP synthesis, NADH and FADH_2_ generation, and eventually disrupts cellular energy homeostasis [57]. ROS produced during PDT can specifically inactivate iron-sulfur cluster-containing enzymes in mitochondrial Complexes I and II, as well as cause carbonylation of TCA cycle dehydrogenases, further exacerbating the energetic deficit, according to mounting evidence in fungal models [58,59].

Similar to the decreased ATP and acetyl-CoA levels seen in our study, other photodynamic studies on Candida albicans have documented fast mitochondrial membrane depolarization and NAD(P)H depletion following photosensitizer activation [60].

### 3.6. Effect of PDT Combined with CDs on the Sensitivity of A. alternata to Insecticide

**Figure 7:**
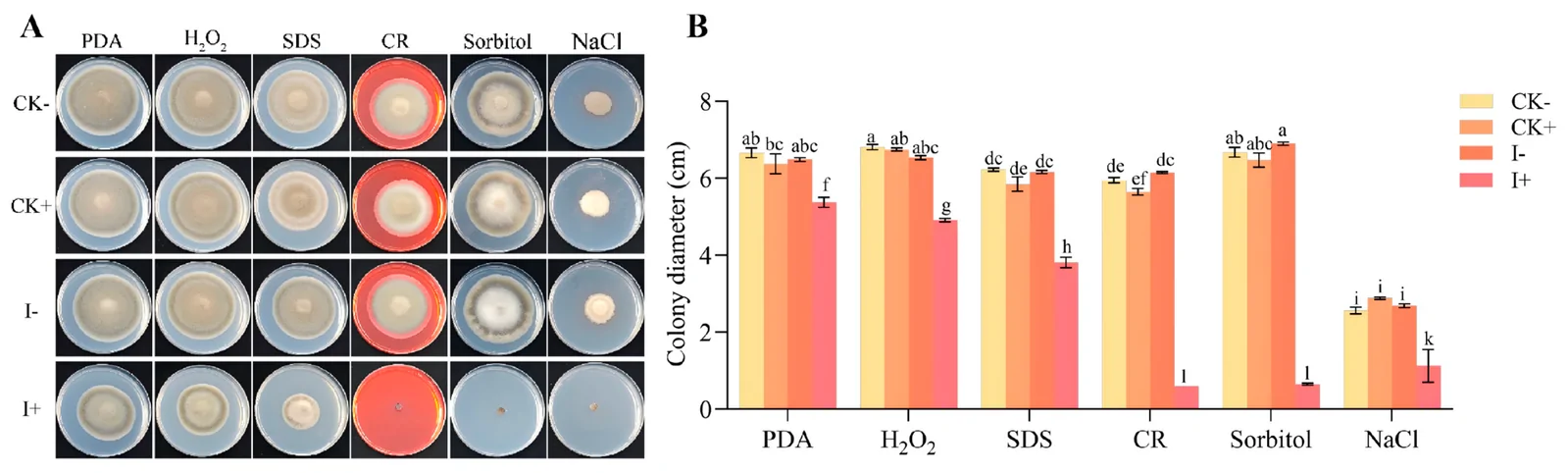
Effect of CDs on the sensitivity of *A. alternata* to exogenous stress. Colony patterns under exogenous stress from H_2_O_2_, SDS, CR, sorbitol, and NaCl (**A**); colony diameters under different stress conditions (**B**). Data are presented as mean ± SD (n = 3). Different letters indicate significant differences (*p* < 0.05, one-way ANOVA followed by Tukey’s HSD test).

To evaluate the sensitivity of *A. alternata* to various stresses following treatment with CDs, the growth of *A. alternata* on agar plates was assessed using an exogenous addition method. On day 5 of cultivation, compared with the CK− group, there were no significant differences among the CK+, I−, and I+ groups on PDA medium or under H_2_O_2_, SDS, CR, sorbitol, and NaCl stress conditions (Figure 7). However, the I+ group treated with PDT combined with CDs showed a 19.9% reduction in colony diameter on PDA medium, and decreased by 27.6%, 48.6%, 89.9%, 90.2%, and 72.5%, respectively, under oxidative stress (H_2_O_2_), cell wall synthesis inhibitors (SDS and CR), and osmotic stress (sorbitol and NaCl) (Figure 7). The results indicate that treatment with PDT combined with CDs increases the sensitivity of *A. alternata* to various stresses, with the organism being most sensitive to the cell wall synthesis disruption caused by CR and to the osmotic stress induced by 1 M sorbitol and 1 M NaCl. Studies have shown that an imbalance in the antioxidant system can lead to increased cellular sensitivity to various forms of stress [61]. The ROS imbalance in *A. alternata* caused by the combined PDT and CDs treatment, particularly when H_2_O_2_ was added exogenously, may have further exacerbated sensitivity to oxidative stress, resulting in severely inhibited growth of the fungus. CR binds to β-1,3-glucan and chitin in the fungal cell wall, interfering with their normal cross-linking and thereby disrupting the structural integrity of the cell wall [62]. Under stress induced by this cell wall synthesis inhibitor, *A. alternata* struggles to repair the damaged cell wall, making it difficult for the fungus to grow on agar plates; its growth is similarly severely limited under conditions of additional exogenous osmotic stress. The observed cell wall damage (Section 3.2) is consistent with the increased sensitivity to CR and NaCl, indicating that CDs-PDT impair both osmotic balance and cell wall integrity. The fungal cell wall integrity (CWI) signaling pathway, which controls the synthesis of chitin and β-glucan in response to external insults, is known to be disrupted by ROS accumulation in addition to direct structural disruption [63]. This disruption probably intensifies the inhibitory effects of cell wall-perturbing agents like CR and SDS. Additionally, the I+ group’s significantly lower tolerance may be explained by oxidative inactivation of upstream sensors or mitogen-activated protein kinases in the high osmolarity glycerol (HOG) pathway, which mediates adaptation to osmotic stress (sorbitol and NaCl) and is extremely sensitive to intracellular redox imbalances [64]. Depleted antioxidant reserves (GSH depletion, decreased CAT activity) are probably the causes behind increased sensitivity to oxidative stress (H_2_O_2_), resulting in a synergistic oxidative stress impact. All these findings support earlier findings that oxidative damage caused by PDT predisposes fungal cells to various environmental stressors by disrupting their adaptive signaling networks [65].

**Figure 8:**
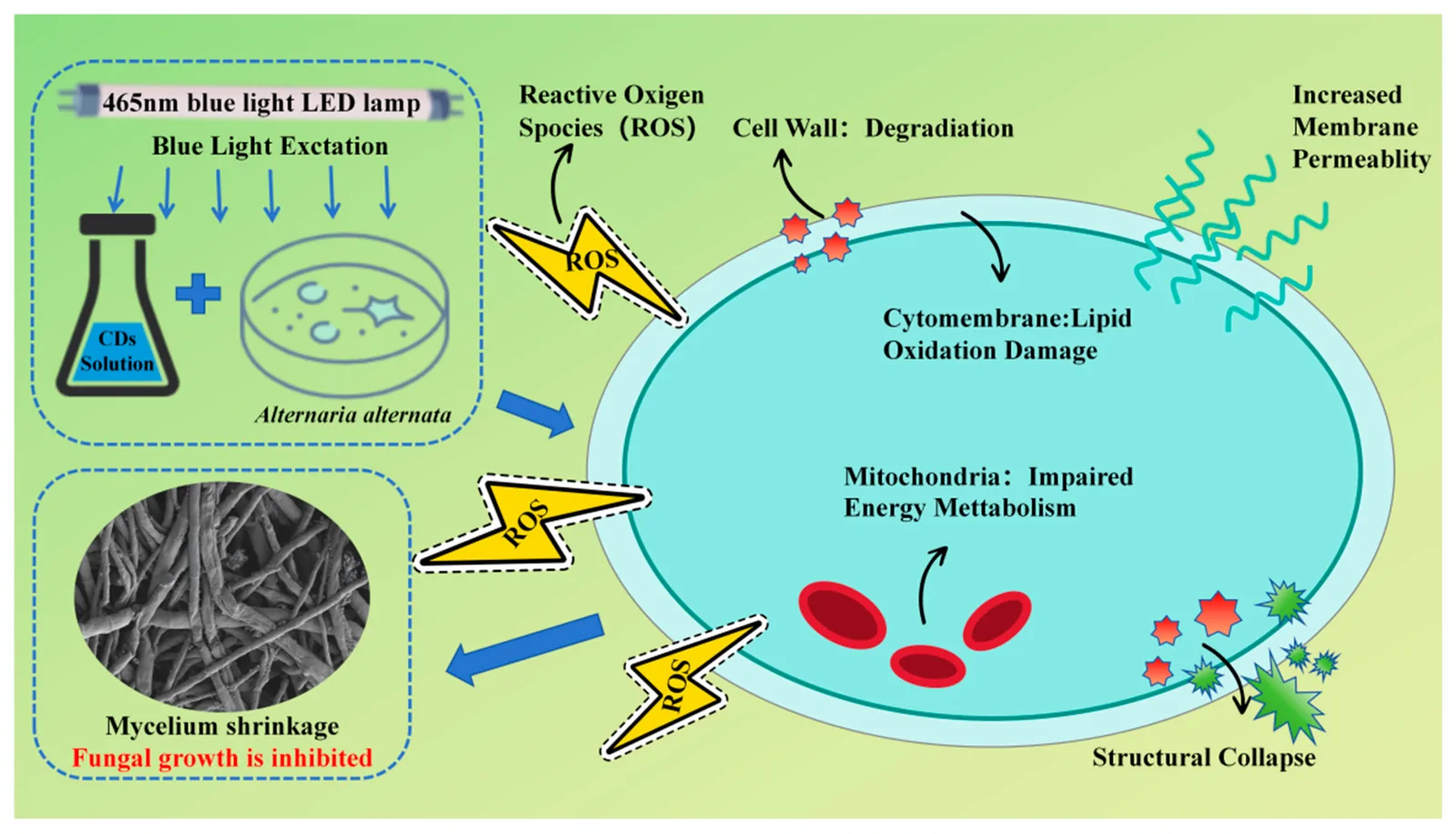
Schematic diagram illustrating the cellular mechanisms underlying the inhibitory effects of CDs on *A. alternata*. CDs-PDT generates ROS through Type I (O_2_^−^, OH) and Type II (^1^O_2_) reactions, causing cell wall/membrane damage, antioxidant system collapse, mitochondrial dysfunction, energy depletion, and increased stress sensitivity, ultimately leading to fungal cell death.

Based on the collective findings, we propose a multi-target mechanism for CDs-PDT antifungal activity against *A. alternata* (Figure 8). Upon blue LED irradiation, CDs generate abundant ROS (primarily H_2_O_2_ and O_2_^−^) through both Type I and Type II photochemical reactions. These ROS directly attack the cell wall (oxidizing chitin and glucan frameworks) and cell membrane (inducing lipid peroxidation), compromising structural integrity. Concurrently, intracellular ROS levels surge, overwhelming the antioxidant defense system despite compensatory upregulation of SOD and GPX. CAT inactivation and GSH depletion create a positive feedback loop, exacerbating oxidative damage. Mitochondrial dysfunction (evidenced by reduced ATP and acetyl-CoA levels, and NADH/NAD^+^ ratio elevation) impairs energy metabolism, depriving cells of the energy required for repair and survival. The cumulative damage renders *A. alternata* hypersensitive to external stresses (oxidative, osmotic, cell wall-damaging), ultimately leading to cell death. This multi-target mechanism explains the potent antifungal efficacy of CDs-PDT and its advantage over conventional single-target fungicides.

## 4. Conclusions

This study systematically demonstrated the potent inhibitory effect of CDs-PDT against *A. alternata* through multifaceted mechanisms. When CDs and blue LED light were applied separately to *A. alternata*, their antifungal effects were limited; however, the photodynamic effect produced by the combination was the primary factor responsible for the pronounced antifungal activity. Mechanistically, CDs-PDT treatment induced disruption of cell wall integrity, damage to cell membrane structure, an intracellular ROS burst through both Type I and Type II photochemical reactions, collapse of the antioxidant defense system, and impaired energy metabolism. Furthermore, the disruption of ROS homeostasis exacerbated fungal sensitivity to various stresses, particularly cell wall disruption and osmotic stress. Despite these advances, critical knowledge gaps remain regarding ROS contributions, molecular targets of oxidative damage, structure–activity relationships of CD properties, and translational challenges for commercial deployment. Future studies should employ multi-omics approaches to elucidate the molecular regulatory network underlying CDs-PDT antifungal activity. Additionally, investigation of CDs-PDT effects on mycotoxin production (ATX II, AOH, AME) would be highly relevant for food safety applications. Ultimately, this technology holds immense potential for green postharvest preservation of agricultural products, offering a sustainable alternative to conventional chemical fungicides. From an application perspective, CDs-PDT-based treatment may overcome long-standing drawbacks of synthetic fungicides, including chemical residue, pathogen resistance and environmental pollution, thereby providing a promising eco-friendly strategy for industrial-scale fruit and vegetable preservation. Future multi-omics and mycotoxin-related investigations are therefore critical to bridge the gap between laboratory-scale mechanistic research and commercial implementation of this photodynamic technology.

## Data Availability

The original contributions presented in the study are included in the article; further inquiries can be directed to the corresponding author.
